# A Functional Human-on-a-Chip Autoimmune Disease Model of Myasthenia Gravis for Development of Therapeutics

**DOI:** 10.3389/fcell.2021.745897

**Published:** 2021-11-22

**Authors:** Virginia M. Smith, Huan Nguyen, John W. Rumsey, Christopher J. Long, Michael L. Shuler, James J. Hickman

**Affiliations:** ^1^Hybrid Systems Lab, NanoScience Technology Center, University of Central Florida, Orlando, FL, United States; ^2^Hesperos, Inc., Orlando, FL, United States

**Keywords:** acetylcholine receptor, neuromuscular junction (NMJ), myasthenia gravis, autoantibodies, microphysiological systems, human-on-a-chip, induced pluripotent stem cells (iPSCs), disease pathology

## Abstract

Myasthenia gravis (MG) is a chronic and progressive neuromuscular disease where autoantibodies target essential proteins such as the nicotinic acetylcholine receptor (nAChR) at the neuromuscular junction (NMJ) causing muscle fatigue and weakness. Autoantibodies directed against nAChRs are proposed to work by three main pathological mechanisms of receptor disruption: blocking, receptor internalization, and downregulation. Current *in vivo* models using experimental autoimmune animal models fail to recapitulate the disease pathology and are limited in clinical translatability due to disproportionate disease severity and high animal death rates. The development of a highly sensitive antibody assay that mimics human disease pathology is desirable for clinical advancement and therapeutic development. To address this lack of relevant models, an NMJ platform derived from human iPSC differentiated motoneurons and primary skeletal muscle was used to investigate the ability of an anti-nAChR antibody to induce clinically relevant MG pathology in the serum-free, spatially organized, functionally mature NMJ platform. Treatment of the NMJ model with the anti-nAChR antibody revealed decreasing NMJ stability as measured by the number of NMJs before and after the synchrony stimulation protocol. This decrease in NMJ stability was dose-dependent over a concentration range of 0.01–20 μg/mL. Immunocytochemical (ICC) analysis was used to distinguish between pathological mechanisms of antibody-mediated receptor disruption including blocking, receptor internalization and downregulation. Antibody treatment also activated the complement cascade as indicated by complement protein 3 deposition near the nAChRs. Additionally, complement cascade activation significantly altered other readouts of NMJ function including the NMJ fidelity parameter as measured by the number of muscle contractions missed in response to increasing motoneuron stimulation frequencies. This synchrony readout mimics the clinical phenotype of neurological blocking that results in failure of muscle contractions despite motoneuron stimulations. Taken together, these data indicate the establishment of a relevant disease model of MG that mimics reduction of functional nAChRs at the NMJ, decreased NMJ stability, complement activation and blocking of neuromuscular transmission. This system is the first functional human *in vitro* model of MG to be used to simulate three potential disease mechanisms as well as to establish a preclinical platform for evaluation of disease modifying treatments (etiology).

## Introduction

**FIGURE 1:**
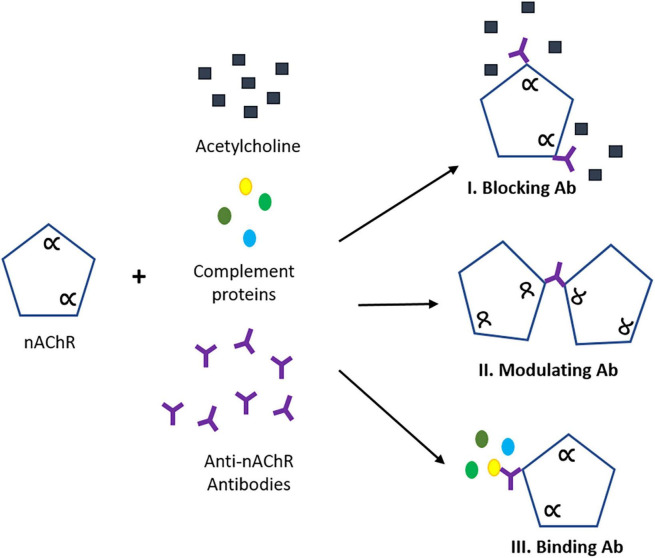
Pathogenic mechanisms of anti-nAChR antibody (Ab). (I) Blocking abs interfere with ACh binding site interrupting neuromuscular transmission. (II) Modulating Abs that bind to two nAChRs causing receptor endocytosis leading to AChR degradation and reduction of expression. (III) Binding Abs that complex with a distinct location on the nAChR, triggering the activation of the complement cascade and subsequent formation of the membrane attack complex (MAC). Scheme adopted from Conti-Fine (Conti-Fine et al., 2006). The alpha (α) refers to the alpha subunit(s) on the nAChR.

Myasthenia gravis (MG) is a rare autoimmune disease that affects intra- and extracellular proteins involved in the neuromuscular junction (NMJ), impairing signal transduction (Lindstrom, 2000; Gilhus and Verschuuren, 2015; Gilhus, 2016). The classic clinical feature of MG is muscle weakness which becomes more severe after repetitive muscle contractions over time. Although most patients will initially develop oculobulbar muscle weakness, 85% of the cases will progress to generalized MG (non-ocular muscle) (Kerty et al., 2014) with antibodies directed against the nicotinic acetylcholine receptor (nAChR; Meriggioli and Sanders, 2009, 2012). The nAChR is a pentameric transmembrane protein consisting of two α1 subunits, one β1 subunit, one δ subunit and either one γ subunit (in embryonic AChR) or one ε subunit (in adult AChR). Due to the heterogeneous nature of anti-nAChR antibodies, the epitope location on the AChR surface is crucial in the disease pathogenesis. Generally, anti-nAChR antibodies (Abs) have three pathogenic mechanisms: (I) direct blocking of the AChR, (II) modulation of the receptor through crosslinking of two AChRs leading to internalization and subsequent degradation, or (III) binding to the receptor at a different antigenic site and activating the complement cascade (Kang et al., 2015; Figure 1). We describe here a system to simulate these three mechanisms. Currently, one of the most common tests to detect autoantibodies against AChR is by a radioimmunoprecipitation assay (RIA) to quantify the patient antibody titer by the precipitation of the specific binding of radiolabeled α-bungarotoxin (I^125^-BTX) to the α1 subunits of AChR (Leite et al., 2010; Zisimopoulou et al., 2013). For antibodies that directly block the ACh-binding site, there will be a decrease in I^125^-BTX-AChR complexes, resulting in a rare but acute inhibition of function (Whiting et al., 1983; Lang et al., 1988; Burges et al., 1990; Bufler et al., 1996). The subtype of nAChR Ab present in seropositive patients has rarely been related to clinical severity (Howard et al., 1987), although, limited evidence suggests blocking and modulating antibodies have been found to have unfavorable prognosis (Kang et al., 2015). There is a need for a more specific human-based platform that could work synergistically with the current detection assays to understand the pathology of MG as well as to develop therapeutic interventions.

Until recently, autoimmune diseases have been studied using cell-based assays to assess antigen activation, cellular interaction, and migration and in animal models by inducing the disease pathophysiology (Giese and Marx, 2014; van de Stolpe and Kauffmann, 2015). However, both approaches lack clinical disease relevance due to the simplistic nature of the assays and intrinsic differences between clinical and experimental autoimmune MG (EAMG) immune responses. These differences further limits understanding of the underlying disease mechanisms. Specifically, in EAMG models, animals are immunized with foreign AChRs and later develop MG-like symptoms providing a potential method to understand MG and the mechanisms involved (Patrick et al., 1973; Losen et al., 2015). Although animal studies have led to advances in MG disease understanding while mimicking symptomatic parameters (Christadoss et al., 2000) and evaluating new immunotherapies (Baggi et al., 2012), the mouse/rat immune system is fundamentally different than a human. Additionally, some EAMG experimental parameters can lead to severe animal suffering and death resulting in skewed statistical analysis (Mantegazza et al., 2016). There is a need to establish new models to create ethically sound experiments that can translate to human disease.

Recently, human-based *in vitro* platform systems have been used to model disease neuronal pathogenesis, develop novel therapeutics, reduce animal experimentation, and improve disease understanding (Mantegazza et al., 2016). These systems use representative human cell cultures utilizing healthy and diseased subjects from primary cells cultured directly from human biopsies (Bellin et al., 2012; Sato and Clevers, 2013) as well as induced pluripotent stem (iPS) and adult stem cells from patients that can be differentiated into varying cell types (Takahashi et al., 2007; Bellin et al., 2012; Sato and Clevers, 2013). Several advances have been made using human-based *in vitro* systems to study diseases that affect the neuromuscular junction (NMJ; Park et al., 2013; Zahavi et al., 2015; Santhanam et al., 2018; Bakooshli et al., 2019). These *in vitro* systems include serum free, functionally relevant mimics of the human NMJ (Santhanam et al., 2018; Guo et al., 2020a, b) that allow crosstalk of the two different cell types (skeletal muscle and motoneurons), real-time functional analysis of direct and indirect stimulation of skeletal muscle and spatially controlled dosing of compounds to provide improved systems to understand human disease mechanisms as well as to study therapeutic options.

**FIGURE 2:**
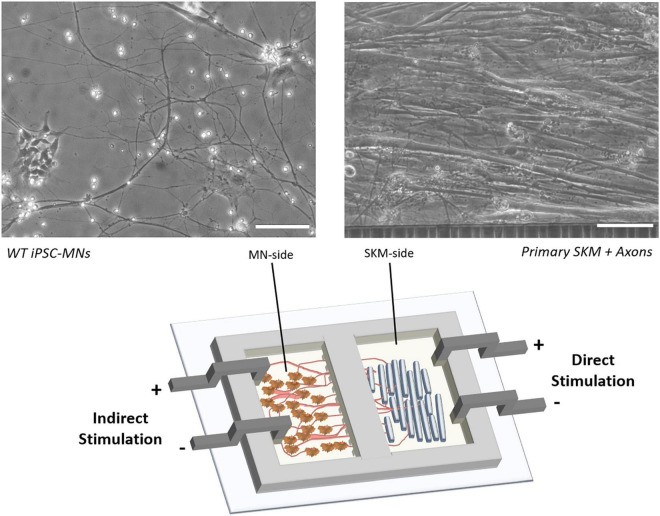
Schematic of NMJ chamber with primary SKM innervated by WT iPSC-MNs and electrically stimulated by electrodes for either direct (SKM-side) or indirect (MN-side) stimulation. Phase images indicate the morphology of co-culture on SKM innervated by axons progressing through tunnels (right) and WT iPSC derived MNs (left). Scale bars = 100 microns.

Here we report the use of a human functional NMJ model system treated with a commercially available antibody against nAChR to mimic the MG disease phenotype. The NMJ chambers were used for the co-culture of primary human skeletal muscle (SKM) and wild-type human induced-pluripotent stem cell (hiPSC)-derived motoneurons (MNs; Figure 2). Separation of the cultures by microtunnels allowed for axonal progression through the tunnels to innervate the myotubes, creating complete electrical and chemical stimulation isolation and enabled selective dosing of compounds to each chamber. Historically, synaptic connections in NMJs have been confirmed through ICC with co-localization of pre- and postsynaptic staining of neurofilament or synaptic protein and bungarotoxin (Arnold et al., 2014; Sleigh et al., 2014; Falk et al., 2015). We have also met this standard in our current NMJ systems based on ICC in our previous publications (Guo et al., 2011; Smith et al., 2013; Santhanam et al., 2018; Guo et al., 2020b). However, our functional NMJ model (indirect stimulation of MNs and real-time contraction of skeletal muscle) is now the best indicator of synaptic connection, validates the co-localization of ICC, and ultimately advances the current standard.

To mimic the disease phenotype and pathology of MG, a commercially available polyclonal antibody against the nAChR was dosed on the muscle side and functional readouts of NMJ stability and fidelity were analyzed. These two parameters were chosen based on the disease mechanisms of MG and reliable clinical measurements used to assess the MG disease pathology, such as muscle weakness after exercise. Since antibody induced effects can result in temporary or permanent reduction of nAChRs, NMJ stability then NMJ number is evaluated to focus on the ability of an NMJ to be maintained after strenuous exercise (indirect stimulation of the SKM at 0.33, 0.5, 1.0, and 2.0 Hz). Since many electromyography (EMG) labs use high frequency stimulation (∼3 Hz) as a standard for NMJ testing, the synchrony of contractions (fidelity) were monitored at 2.0 Hz (the historic frequency used in our systems). A concentration dependent dose response curve was determined using the anti-nAChR IgG antibody to establish the optimal dosing to elicit a functional deficit in NMJ stability. Complement activation in the presence of antibody caused a functional, dose-dependent loss of NMJs. Additionally, complement protein deposition can be initiated, as confirmed by immunocytochemistry, to reduce the number of contracting myotubes utilizing direct and indirect skeletal muscle (SKM) fidelity. Thus, the described MG model provides a diagnostic platform sensitive to the pathogenic mechanisms of MG and more importantly, can be utilized for the development of potential patient-specific treatments. These data indicate that this human-based NMJ system can mimic key aspects of antibody- and complement-mediated MG pathology and can be used to evaluate disease modifying treatments (DMTs) of MG.

## Materials and Methods

### Study Design

To determine the dose-response of the NMJ system to anti-nAChR IgG, dosages were selected across a range of more than 2 orders of magnitude initially to identify the upper and lower plateau values, as well as the approximate range in which the IC_50_ occurred. The range of a single order of magnitude around the IC_50_ was further evaluated in more detail across the range with dosages split approximately equally in the log scale. Regression analysis using least square multiple regression to a sigmoidal equation with upper and lower plateau values, Hill coefficient, and IC_50_ was performed to numerically determine the response shape. Sample sizes for the functional experiments were determined using estimated sample variance from preliminary experiments, such that statistical analysis would detect differences among treatment groups of 50% with 80% power and type I error rate of 0.05 (α = 0.05). For the five main conditions (WT, 2 μg/mL anti-nAChR Ab, 2 μg/mL non-specific IgG, 0.05% hCS, and the combination of 2 μg/mL anti-nAChR Ab + 0.05% hCS), a minimum of eight replicates were assessed from at least two separate experiments (*N* = 2). Following data collection, treatment groups were statistically compared using one-way ANOVA followed by Fisher’s LSD with α = 0.05.

### Fabrication and Assembly of the Neuromuscular Junction Chamber Co-culture System

Neuromuscular junction chambers were cast using polydimethylsiloxane (PDMS) (Sylgard-184, Dow Silicones Corp., Cat. No. 2065622) at a 10:1 (base: curing agent) ratio. Once the base and curing agent were properly mixed, the PDMS was poured onto the SU-8 wafer mold. The fabrication of the SU-8 wafers used for the casting of the NMJ chambers were prepared using previously described methods (Santhanam et al., 2018). The wafer was then placed in the oven at 80°C overnight. After curing, the PDMS NMJ chambers were cut from the mold and soaked in 70% isopropyl alcohol (IPA) overnight to sterilize and remove any uncured monomers. The chambers were rinsed with additional 70% IPA and dried overnight in a biosafety cabinet in preparation for assembly.

Glass coverslips (VWR, Cat. No. 48366067) were plasma treated using a Harrick Plasma cleaner (model PDC-32G) with high-purity oxygen gas for 2 min at 750 mTorr. The coverslips were then sterilized using 70% ethanol and allowed to air dry. Sterilized NMJ chambers were sealed onto the glass coverslips by gently pressing the PDMS chambers on top of the plasma treated glass coverslips.

### Surface Coating

Once sealed, the skeletal muscle (SKM) side of the NMJ chamber system (Santhanam et al., 2018) was coated with 60 μg/mL of rat tail collagen I (Gibco, Cat. No. A10483-01) in 1X phosphate buffer solution (PBS). The motoneuron (MN) side was coated with 3 μg/ml of laminin solution (Invitrogen, Cat. No. 23017-015) in 1X PBS. The chambers were left to incubate at room temperature for 2 h. The collagen coating solution was then removed and rinsed with sterile 1X PBS. Serum-free adult growth medium (AGM) was added to the muscle side and the systems were placed in a 4°C refrigerator overnight. The laminin coating solution was removed after overnight refrigeration and human motoneuron (hMN) medium was added to the MN-side. The chambers were incubated at 37°C, 5% CO_2_ for 1 h prior to skeletal muscle plating.

### Culture of Human Skeletal Muscle Myoblasts

Primary human skeletal muscle myoblasts (Lonza, Cat. No. CC2580; lot 483427) were expanded once and used for all experiments. Myoblasts were seeded at a density of 300 cells/mm^2^ in the NMJ chambers. At 70–80% confluency, the cultures were switched to differentiation medium (NbActiv4, BrainBits LLC, Cat. No. Nb4-500) to induce myogenesis. A one-third medium change with NbActiv4 was conducted every 2 days until day 12 of testing.

### Culture of Human Induced Pluripotent Stem Cell-Derived Motoneurons

Human motoneurons were derived by differentiation of human iPSCs isolated from a healthy subject (Coriell Institute, Cat. No. ND41865). Wild-type human motoneurons used in this study were differentiated from iPSCs (cell passaging number 6–10) using previously established protocols (Santhanam et al., 2018) and plated on the MN-side of the NMJ chamber system 4 days after myoblast differentiation. Motoneurons were seeded at a density of 1,500 cells/mm^2^. A one-third medium change in the NMJ chambers was conducted every 2 days until day 12 of testing.

### Anti-nicotinic Acetylcholine Receptor Antibody and Human Complement Serum Dosing

After 12 days post-differentiation of SKM, systems were dosed with either an antibody against the nAChR α1 (Abcam, Cat. No. ab221868), or a non-specific IgG1 antibody (Calbiochem, Cat. No. NE1023), and human complement sera (Sigma-Aldrich, Cat. No. S1764-1ML). Systems were dosed on the SKM side with anti-nAChR antibody only (0.01–20 μg/mL), non-specific IgG1 (2.0 and 10 μg/mL), human complement sera only (0.05%), or both anti-nAChR antibody and human complement sera at 2 μg/mL and 0.05%, respectively. After dosing, systems were incubated at 37°C, 5% CO_2_ for 3 h prior to testing.

### Electrophysiological Testing

Prior to evaluation of NMJ physiology (i.e., SKM contraction in response to MN stimulation), electrical isolation of the MN-side and SKM-side was determined to ensure no electrical leakage between compartments. Briefly, chlorinated silver electrodes connected to an epithelial voltohmmeter (EVOM) were submerged into the NMJ chambers. The current between the 2 compartments was recorded. Cultures with a resistance value <5000 Ω were excluded from further electrical testing due to risk of electrical leakage that could interfere with isolated skeletal muscle contraction. Systems that met the criteria for electrophysiological testing were transferred onto a temperature controlled heated stage set at 37°C for testing. Cultures were imaged using an upright Zeiss Hal 100 microscope and videos were recorded using a Hamamatsu digital camera (Model C8484-056) with LabVIEW software. Throughout the stimulations, myotube contractions were monitored by live video recordings of pixel differentials.

Single pulses of 4 V were manually delivered to either the SKM-side (direct stimulation) or the MN-side (indirect stimulation) and the pixel subtraction generated (pixel differentials) was used to visually quantify the total number of contracting myotubes in six different frames. The number of contracting SKMs under indirect stimulation (number of NMJs pre-stimulation) was collected within 2 min. One myotube is selected (Region of interest – ROI) from each chamber system and undergoes electrical stimulation (see stimulation protocol in Table 1) with pulses of increasing frequency (0.33, 0.5, 1.0, and 2.0 Hz) under indirect and direct stimulation. Skipping during the duration of each stimulation frequency was recorded. The number of synchronized contractions (N_*s*_) at a given pulse was divided by the number of stimulation pulses within a set duration (N_τ_) to give NMJ fidelity:


(1) $$\frac{{\text{N}}_{\text{s}}}{{\text{N}}_{\tau }}=\mathrm{NMJ}\,\mathrm{Fidelity}$$

**TABLE 1:** Stimulation protocol for direct and indirect stimulation of SKM.

**Stimulation protocol**	
**Frequency (Hz)**	**Duration (s)**
0.33	60
0.5	40
1.0	20
2.0	10

After the completion of the stimulation protocol, the total number of contracting SKM under indirect stimulation (NMJs) were recorded again using manual stimulation and visual assessment of the pixel differentials. To measure the stability of NMJs per system, the total number of NMJs counted post-stimulation divided by the total number of NMJs pre-stimulation was recorded as a percentage. Each condition was subjected to this stimulation process once in at least four different systems.

### Immunocytochemistry

#### General Immunocytochemistry

Monocultures of SKM at day 12 of differentiation were fixed using 4% paraformaldehyde for 10 min and washed twice with 1X PBS (Life Technologies). Cells were blocked using donkey blocking buffer (5% donkey serum, 5% bovine serum albumin, 1X PBS) for 1 h at room temperature. The cells were then incubated with primary antibodies overnight at 4°C. After overnight incubation, the primary antibody was removed, and the cells were washed with 1X PBS for 5 min per wash. The secondary antibodies were incubated for 2 h in the dark at room temperature. The secondary antibodies were removed, and the cells were washed three times with 1X PBS for 5 min each wash. The cells were then incubated with 4′,6-Diamidino-2-Phenylindole (DAPI) (Thermo Fisher Scientific, Cat. No. 62248) for 5 min. The DAPI solution was removed, and the cells were washed three times with 1X PBS for 3 min each wash. The coverslips were then mounted using a ProLong^®^ Gold Antifade Mountant (Thermo Fisher Scientific, Cat. No. P36930). Fluorescence imaging was performed using UltraView^TM^ spinning disk confocal microscope (PerkinElmer) with an Axio Observer Z1 (Carl Zeiss) stand utilizing 20x/0.75 air and 40x/0.75 air objectives. Volocity software was used to process Z-stack projections of scanned images.

#### Complement Deposition Studies

The ICC protocol was followed with the following modifications: Before fixing the SKM monocultures, cells were either incubated at 37°C, 5% CO_2_ for 3 h with a combinatory dosing of anti-nAChR Ab (Abcam, Cat. No. ab221868, 2 μg/mL) and human complement sera (Sigma-Aldrich, Cat. No. S1764-1ML, 0.05%) or single dosing of human complement sera at varying dilutions (0, 0.1, 1.0, and 10%). Cultures were rinsed with 1X PBS prior to fixing and blocking. The primary antibody used to detect complement deposition was mouse anti-C3/C3b (Abcam, Cat. No. ab11871, 1:500 dilution in blocking buffer). The corresponding secondary antibody (Life Technologies, Cat. No. A10037, anti-mouse-568, 1:250 dilution in blocking buffer) and Alexa Fluor 488 conjugated α-bungarotoxin (Life Technologies, Cat. No. B13422, 1:100 dilution in blocking buffer) were then incubated for 2 h in the dark at room temperature.

#### Endogenous and Diseased Receptor Internalization Studies

The ICC protocol was followed with the following modifications: prior to SKM fixing, monocultures were either incubated at 37°C, 5% CO_2_ for 3 h with anti-nAChR Ab (Abcam, Cat. No. ab221868, 2 μg/mL or 10 μg/mL) for diseased receptor internalization studies or with SKM medium for the endogenous internalization control. For each condition, the monocultures were either permeabilized with 0.1% Triton X-100 or not for 15 min before blocking for 1 h. The endogenous internalization control cultures (pre-incubation with medium) were fixed and then incubated for 3 h with anti-nAChR Ab (Abcam, Cat. No. ab221868, 2 μg/mL). The corresponding secondary antibodies were then added to all cultures.

Using ImageJ, myotubes were selected from at least ten fluorescence images using a freeform tool and measured against non-fluorescent backgrounds to obtain values for integrated density, area of selected cell, and mean fluorescence of background readings to calculate the corrected total cell fluorescence (CTCF) of cells +/− triton.


(2) CTCF=Integrateddensity-(AreaofselectedcellxMeanfluorescenceofbackgroundreadings)

To determine the nAChR internalization, the change in *CTCF* of SKM +/− triton was determined by Eq. 3 below (Howard and Sanders, 1980).


(3) $$nAchRInternalization=CTCF_{+t\,r\,i\,t\,o\,n}-CTCF_{-t\,r\,i\,t\,o\,n}/C\,T\,C\,F_{+t\,r\,i\,t\,o\,n}*100$$

## Results

### Anti-nicotinic Acetylcholine Receptor Antibodies Affect Neuromuscular Junction Stability and the SKM Excitation-Contraction Coupling Mechanism in a Dose-Dependent Manner

Anti-nAChR antibodies reduce NMJ stability and the SKM excitation-contraction coupling mechanism in a dose-dependent manner. Most MG autoantibodies are directed against the nAChR with more than 50% against the α subunit, specifically the main immunogenic region (MIR) in both innate and experimental autoimmune MG (EAMG; Tzartos et al., 1998; Luo et al., 2009). These nAChR autoantibodies are mainly of the IgG1 and IgG3 subclasses (Lefvert et al., 1981; Rodgaard et al., 1987) and can initiate varying disease pathogenicity depending on the epitope location (Tzartos et al., 1998). To establish the effect of the anti-nAChR IgG antibody on the NMJ *in vitro* system, a concentration curve against NMJ function was generated to evaluate NMJ stability based on criteria described in our previous publications (Santhanam et al., 2018; Guo et al., 2020b) and in the materials. In brief, after a 3 h incubation period a single field electrical stimulation was applied to the MN side of the chamber and the total number of NMJs before and after the stimulation protocol was applied, were visually assessed. This was represented as a percentage of NMJ stability. Since anti-AChR Abs can induce both direct blocking and internalization of receptors, potentially causing variable NMJ numbers at any given time, the stability of an established NMJ was evaluated instead.

**FIGURE 3:**
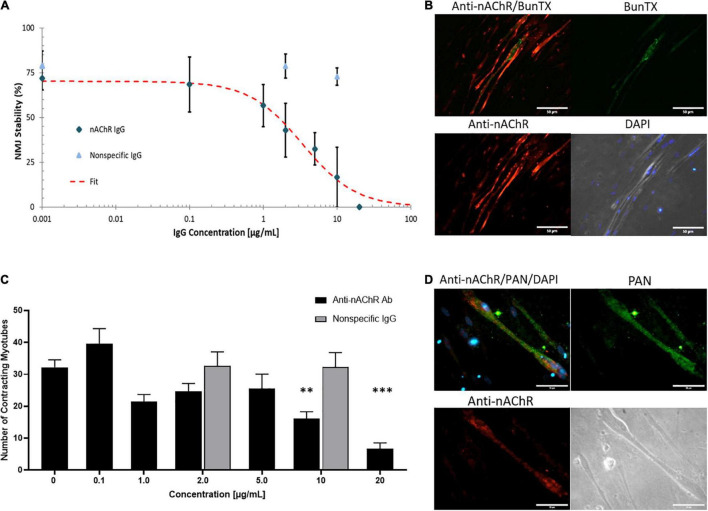
Anti-nAChR IgG effects on the NMJ. **(A)** A dose response curve established from NMJ stability of SKM dosed with the anti-nAChR Ab (diamond) from 0 to 20 μg/mL resulting in an IC50 of 3.4 μg/mL (*N* = 2, 3–10 replicates). A non-specific IgG antibody (triangle) did not significantly alter NMJ stability at 2 and 10 μg/mL (*N* = 2, 7–8 replicates). **(B)** ICC images of SKM revealing the antibody-antigen complex with the binding of anti-nAChR Ab (red channel) to the cell surface near the endplate marked by BunTX (green channel), overlay of red and green channels (upper left) and phase image with DAPI (lower right). Scale bars = 50 microns. **(C)** The number of contracting SKM under direct stimulation at dosing concentrations from 0 to 20 μg/mL (*N* = 2, 3–10 replicates). Statistics: One-way ANOVA followed by Dunnett’s test (alpha = 0.05) ^∗∗^*p* < 0.020, ^∗∗∗^*p* < 0.002. **(D)** ICC images of SKM with anti-nAChR Ab staining (red channel), sodium channel (Pan, green channel), overlay of red and green channels (upper left) and phase image on lower right. Scale bars = 50 microns.

The muscle-side of the NMJ systems was dosed with a commercially available antibody against nAChR for 3 h and an IC_50_ of 3.4 μg/mL was generated as a function of change in NMJ stability (Figure 3A). A non-specific IgG1 antibody was also dosed for 3 h as a negative control and revealed no functional deficits at concentrations below and above the established IC_50_ of the antagonistic antibody (Figure 3A). Dose-dependent reduction in NMJ stability with anti-nAChR IgG compared to no effect from the negative control, substantiated the specificity of the anti-nAChR antibody in the *in vitro* NMJ system. The formation of the antibody-antigen complex on the SKM was confirmed by ICC with dual BunTX and anti-nAChR Ab staining (Figure 3B). The specific IgG anti-nAChR Ab binds near the cell surface endplate marked by BunTX. Since distinguishing the mechanism of antibody binding as direct blocking, antigenic modulation, or complement activation is difficult, the direct effects of anti-nAChR Abs on muscle contraction was investigated (Figure 3C). Prior to NMJ testing, direct stimulation of the skeletal muscle (SKM) was initiated and the number of contracting myotubes were measured to establish baseline function. Upon dosing with the antagonist antibody into the muscle chamber, no significant change in the number of contracting myotubes was observed at concentrations equal to or less than 5 μg/mL. However, a significant decrease (*p* = 0.0145 and *p* = 0.0018) in the number of contracting myotubes under direct stimulation at 10 and 20 μg/mL dose of anti-nAChR Ab compared to no dose (0 μg/mL) was observed (Figure 3C). There was no significant change at low or high concentrations (2 and 10 μg/mL, respectively) of non-specific IgG compared to no dose. This functional effect had not previously been described in EAMG or clinical studies, however, due to the compartmentalized nature of the NMJ system, motoneuron independent stimulation or direct stimulation of the muscle can be tested. We speculate that this decrease in functional myotubes was due to a reduction of the number of plasma membrane-bound ion channels involved in the excitation-contraction coupling mechanism. Since the human *in vitro* cultures do not exhibit the structures of the NMJ found *in vivo* where postsynaptic machinery form indentions isolating nAChRs, but instead cluster near diffuse receptors responsible for excitation-contraction machinery, it is possible that endocytosis is not limited to nAChRs. Antigenic modulation would not only cause internalization of surface nAChRs but also decrease the number of neighboring receptors, particularly sodium and calcium channel receptors. To further evaluate this possibility, immunocytochemical analysis of sodium channels (Na_*v*_-PAN) revealed a proximity and abundance of PAN channels near nAChRs on the skeletal muscle (Figure 3D). A loss of sodium channels, and the resultant reduction in sodium current, in MG patients, increasing the threshold for triggering an endplate potential (EPP; Ruff and Lennon, 1998, 2008). Therefore, the reduction of contracting myotubes at higher concentrations of antibody in Figure 3C maybe due, in part, to an increase in internalization and a decrease in neighboring ion receptors critical for eliciting a contractile response.

### Addition of Anti-nicotinic Acetylcholine Receptor Antibodies Increases Receptor Internalization

**FIGURE 4:**
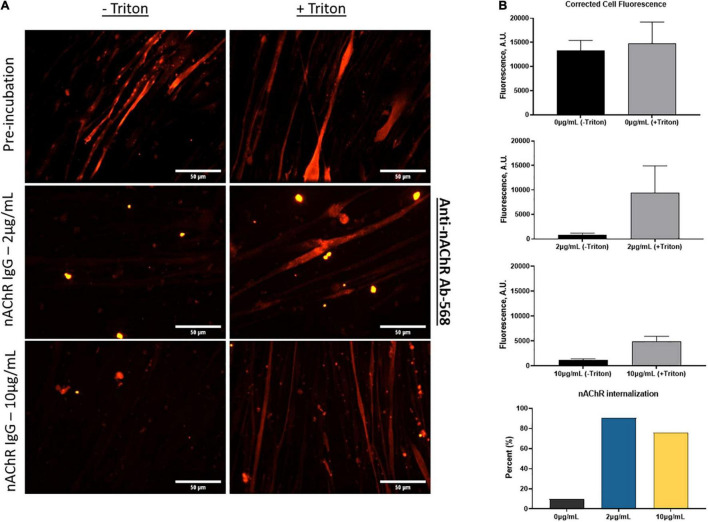
Anti-nAChR Abs induce receptor internalization. **(A)** ICC images of SKM stained for anti-nAChR Ab with and without triton (top panel), incubated with 2 μg/mL or 10 μg/mL anti-nAChR Ab, middle and bottom panels, respectively, and later fixed and blocked with and without triton. Scale bars = 50 microns. **(B)** The corrected total cell fluorescence between the surface receptors (black bars) and internalized receptors (gray bars) for each condition. The percent change in fluorescence is represented as a bar graph for nAChR internalization.

Cells continuously internalize surface receptors by receptor mediated endocytosis. The internalized receptors can undergo a multifaceted array of recycling or degradative pathways. MG patient autoantibodies can induce increased recycling and degradation of AChRs and although significant progress has been made, the mechanism of endocytosis and receptor trafficking is still not well understood (Heinemann et al., 1977; Kao and Drachman, 1977; Drachman et al., 1978; Merlie et al., 1979; Lee et al., 2014). To evaluate whether the anti-nAChR Abs act as modulating antibodies, inducing receptor internalization, the endogenous internalization of nAChRs was first assessed. This evaluation was achieved by fluorescently tagging nAChRs on SKM that were treated with and without a permeabilizing detergent (i.e., Triton X-100). The location of the nAChRs was monitored by labeling the receptors and quantifying the degree of fluorescently tagged receptors that were internalized (with triton) to those on the cell membrane (no triton) using Eq. 2. This steady state of receptor recycling was then compared to internalization due to antigenic modulation by incubation with the anti-nAChR Ab at either 2 or 10 μg/mL. Immunofluorescence analysis of the nAChRs on the SKM surface (no triton) vs. internalized (with triton) is shown in Figure 4A with the corresponding corrected cell fluorescence in Figure 4B (top three panels). The endogenous internalization of the nAChRs was shown to be continuously recycled as the overall fluorescence for both conditions was similar (Figure 4B, top panel). When the anti-nAChR Ab was incubated in the SKM compartment at 2 and 10 μg/mL, there was a near 9-fold and 7-fold increase, respectively, of internalized receptors compared to the pre-incubated SKM (Figure 4B, fourth panel). Surprisingly, incubation with 10 μg/mL anti-nAChR Ab resulted in a lower increase in receptor internalization compared to 2 μg/mL. We propose this observation was due to an increased rate of internalization and degradation, as well as downregulation of the receptor expression. However, further experiments will be necessary to confirm this hypothesis. Increased internalization and degradation would support the decrease in the number of contracting myotubes observed under direct stimulation of SKM at 10 μg/mL (Figure 3C).

### Complement Deposition at the Neuromuscular Junction Significantly Reduces Neuromuscular Junction and SKM Fidelity

**FIGURE 5:**
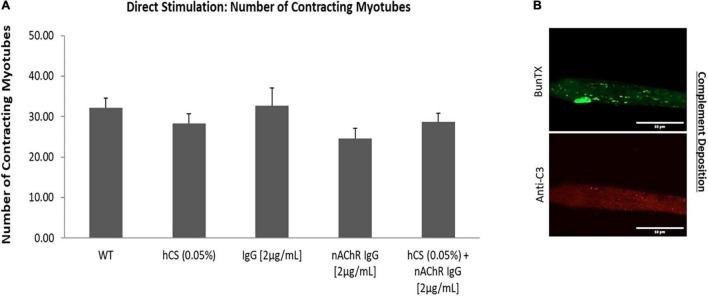
Direct effects of hCS and anti-nAChR on SKM. **(A)** Demonstrates the number of contracting myotubes under direct stimulation after dosing with 0.05% hCS (*N* = 2, 8 replicates), non-specific IgG (2 μg/mL, *N* = 3, 8 replicates), anti-nAChR-Ab (2 μg/mL, *N* = 2, 10 replicates) and a combination of 0.05% hCS and 2 μg/mL anti-nAChR-Ab (*N* = 2, 9 replicates). **(B)** ICC images of SKM incubated with 2 μg/mL of anti-nAChR with 0.05% hCS for 3 h and stained with anti-C3 (red channel) and BunTX (green channel). Scale bars = 50 microns.

The previous experiments determined that the addition of the anti-nAChR Ab can disrupt NMJ stability in a dose-dependent manner. Increased internalization was also confirmed with addition of antibody compared to pre-incubated SKM resulting in a decrease in the number of functional myotubes at higher concentrations of the antibody (>10 μg/mL). To further understand deficits observed in MG, the antibody-induced complement cascade was activated with the addition of anti-nAChR Ab (2 μg/mL) and human complement serum (hCS, 0.05%). To avoid non-specific deposition of complement (Ab-free induced activation), the optimal dosing of complement proteins on the SKM, based on maximal dosing without complement deposition, was confirmed. By quantifying the fluorescence intensity from complement protein 3 (C3) deposition after incubation of hCS on SKM, the optimal dosing of hCS was confirmed to be <0.1% (Supplementary Figure 1). To include functional deficits due to antibody-mediated deposition of complement proteins, a pre-dose of SKM with either 0.05% hCS only or non-specific IgG1 Ab (2 μg/mL) were tested to establish negative controls. To assess functional deficits, the SKM-side of the system was dosed with either 2 μg/mL of anti-nAChR Ab only or the combination of 0.05% hCS and anti-nAChR Ab (2 μg/mL). After direct stimulation of the SKM-side of the chambers, there were no significant effects on the number of contracting myotubes compared to negative controls (Figure 5A). Immunofluorescent analysis of complement activation revealed deposition of complement protein C3 on the SKM after incubation with 2 μg/mL of anti-nAChR Ab and 0.05% hCS (Figure 5B). Deposition confirmed that the antibody can induce activation of the complement cascade, but not reduce the number of contracting myotubes at that combinatory dosage.

**FIGURE 6:**
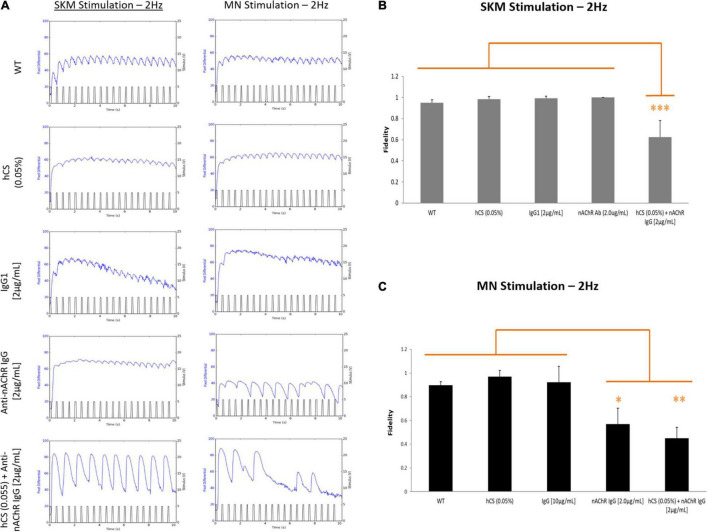
Complement activation reduces SKM and NMJ fidelity. SKM fidelity at 2 Hz under direct and indirect stimulation after incubation with 0.05% hCS (*N* = 2, 6 replicates), non-specific IgG1 (*N* = 3, 7 replicates), anti-nAChR Ab and a combination of 0.05% hCS (*N* = 2, 4 replicates) and 2 μg/mL of anti-nAChR Ab (*N* = 2, 4 replicates). **(A)** Representative stimulation traces generated at 2 Hz from both direct (SKM stimulation) and indirect (MN stimulation) of a myotube under the five conditions. Mean fidelity of peaks under direct **(B)** and indirect **(C)** stimulation. Significant reduction of fidelity with antibody and complement under direct and indirect stimulation and antibody only under indirect stimulation. Statistics: Fisher LSD Method ^∗^*p* < 0.050; ^∗∗^*p* < 0.020; ^∗∗∗^*p* < 0.010.

Neurological tests for MG patients include repetitive stimulation of the nerve at 3 Hz to reveal a decline in the compound muscle action potential (CMAP; Liik and Punga, 2016; Gilhus et al., 2019) and single fiber electromyography (SFEMG) to measure neurological jitters or synchrony between the time of nerve stimulation and muscle contraction (Thornton and Michell, 2012). To mimic the clinical phenotype of neurological jitters (we describe here as fidelity), the muscle was electrically stimulated at 2 Hz and the number of resulting synchronized contractions over the number of stimulation pulses was evaluated (Eq. 1). Representative images of the five conditions under direct and indirect stimulation at 2 Hz is shown in Figure 6A. Once analyzed, the synchrony readouts from each chamber under direct stimulation at 2 Hz demonstrated a ∼60% reduction in fidelity after complement activation with no change observed with the anti-nAChR Ab alone (Figure 6B). Subsequent indirect stimulation of SKM revealed a nearly 57% and 45% reduction in NMJ fidelity in the presence of anti-nAChR Ab and complement activation (hCS + anti-nAChR Ab), respectively, compared to negative controls (Figure 6C). The combinatory deficits found under both direct and indirect stimulation of systems dosed with compliment sera and antibody suggests that we can mimic a more severe pathology when complement is activated.

## Discussion

Here we report the use of our established human *in vitro* NMJ BioMEMs system (Santhanam et al., 2018; Guo et al., 2020b) to dose SKM with a commercially available antibody against nAChR to simulate the three proposed pathogenic mechanisms of MG – blocking, modulation, and binding (Figure 1). Our work was complementary to the clinical phenotype produced from MG patients. In MG, anti-nAChR antibodies reduce the number of AChRs at the endplate resulting in decrease sensitivity to acetylcholine (ACh), impairment of sodium (Na^+^) channels and increased threshold depolarization needed to trigger an action potential (AP), ultimately decreasing the safety factor (SF) for neuromuscular transmission (Ruff and Lennon, 2008). We have shown in our functional NMJ system and subsequent ICC, reduction in NMJ stability and excitation-contraction coupling capability of SKM in the presence of anti-nAChR Abs in a dose-dependent manner. An increase in receptor internalization due to modulation of Abs was also observed compared to pre-dosed SKM. This effect appears to be sensitive to antibody concentration as higher concentrations of antibody had a reduction in recycling and possibly an increase in degradation. Further evaluation of AChR degradation through reduction of AChR clusters will be needed to confirm this speculation. We have also noted that the proximity of sodium (Na^+^) channels to AChR clusters could result in reduction of Na^+^ channels during antibody mediated internalization of AChRs. A reduction of Na^+^ channels and AChRs has been linked to a reduced safety factor (neuromuscular transmission) in MG patients (Ruff and Lennon, 2008) and therefore is a reasonable explanation of the reduction of functional NMJs found in our model system. Complement deposition was also confirmed in the presence of anti-nAChR Ab to the antigen on the cell surface through ICC from complement protein C3 staining near the endplate. Complement activation significantly altered the excitation-coupling capabilities of SKM and NMJ SKM fidelity at 2 Hz. In electromyography (EMG) laboratories, a frequency of 3 Hz is standard for RNS to assess neurological diseases (Zivković and Shipe, 2005; Selvan, 2011; Stålberg et al., 2017). In our fidelity studies, we were able to detect deficits at lower frequency with no significant change below 2 Hz (data not shown). This similarity in frequencies suggests that our *in vitro* systems are translatable to the physical tests measured in clinical studies (Stålberg et al., 2017; Patel and Pobre, 2020). Altogether, this combinatory fidelity readout mimics the clinical phenotype of neurological blocking or “jitters” and suggests binding antibodies with complement activation may have a more persistent disease pathology. Based on these data, we believe the synchronous firing metric is important for mimicking the clinical hallmarks of MG – neurological jitters and reduction of AChRs.

Myasthenia gravis is a disorder caused by specific autoantibodies at the neuromuscular junction. The current methods for MG diagnosis include serological tests for specific antibodies (Sanders et al., 2014; Li et al., 2019) and electrodiagnostic test for repetitive nerve stimulation (RNS) to monitor deficits in neuromuscular transmission (Tim and Sanders, 1994). However, there are varying antibodies that contribute to MG and the identification of the most common Ab, anti-AChR Ab, has not been linked to disease severity (Sanders et al., 2014). Additionally, electrodiagnostic tests are invasive and painful and the results can be misinterpreted with other neuromuscular diseases. Once diagnosed, MG patients are subject to broad-based immunosuppressive treatments (IST) that are relatively effective in regulating symptoms, however, long-term side-effects and susceptibility to life threatening infections are often intolerable for patients (Sanders et al., 2016). The development of more directed therapeutics has helped reduce toxicity and create more fast acting and effective treatments. For example, eculizumab, the first FDA approved treatment for MG, is a monoclonal antibody that prevents the cleavage of C5 and essentially inhibits complement-mediated membrane lysis (Wijnsma et al., 2019). For patients that suffer from binding antibodies that activate the complement cascade, this drug reduces the severity of their symptoms, however, additional IST treatments are required as eculizumab only inhibits terminal complement activation but does control circulating antibodies that can lead to muscle fatigue. Animal models of MG (EAMG) are currently used to analyze MG pathology and study new interventions including induction of peripheral tolerance in the thymus, immunomodulating dendritic cells to induce tolerance and protect from autoimmune diseases, inhibitors of complement activation and RNA interference (Mantegazza et al., 2016). Despite EAMG models reproducing some aspects of the disease pathology, several differences are apparent including the inability to recapitulate the spontaneous disease progression, alterations, and general involvement of the thymus in EAMG. In addition, there are inherent differences between animal and human innate and adaptive immunity including immune system development, activation, and response (Mestas and Hughes, 2004). Therefore, any response in EAMG may not occur in the same way as in humans and therefore should be considered with these differences in mind.

Although our system is the first human MG model system to mimic the three pathogenic mechanisms in a concentration-dependent manner, we understand that our functional model needs further complexity to begin to deduce mechanistic insights. In future studies, we will dose our NMJ systems with MG patient sera and extrapolate the anti-AChR Ab concentration to the NMJ functional readout using our concentration curve. Dosing of MG sera within an NMJ system is not a new concept as rudimentary neuromuscular function has been assessed through optogenetics (MN-side only) and chemical stimulation (Bakooshli et al., 2019; Vila et al., 2019). However, the novelty of our system allows not only for a graded response due to Ab concentration but also the ability to assess complement-induced responses under direct and indirect electrical stimulation. In addition, we will be able to use this MG model system to test current therapeutics for MG and its ability to reverse the patient specific disease phenotype. Furthermore, we understand the limitations of our system and like EAMG models, we will not be able to mimic the contributions of the thymus in MG unless we develop a multi-organ model. We are confident that our human MG system has the potential to be a sensitive mimic of MG pathology and provide a quick and cost-effective platform to evaluate patient-specific treatment and DMTs.

## Data Availability Statement

The raw data supporting the conclusions of this article will be made available by the authors, without undue reservation.

## Author Contributions

VS, JR, and JH designed the experiments. VS and HN conducted the experiments. VS, JR, CL, and JH contributed to data analysis and interpretation. CL contributed to statistics. VS, HN, and JH drafted the original manuscript. VS, CL, JR, MS, and JH edited the manuscript. MS and JH provided funding acquisition and project administration. All authors contributed to the article and approved the submitted version.

## Conflict of Interest

JH has ownership interest and is Chief Scientist and member of the Board of Directors in Hesperos, Inc., which may benefit financially as a result of the outcomes of the research or work reported in this publication. The remaining authors declare that the research was conducted in the absence of any commercial or financial relationships that could be construed as a potential conflict of interest.

## Publisher’s Note

All claims expressed in this article are solely those of the authors and do not necessarily represent those of their affiliated organizations, or those of the publisher, the editors and the reviewers. Any product that may be evaluated in this article, or claim that may be made by its manufacturer, is not guaranteed or endorsed by the publisher.
