# Supplementary figures and images for: A Functional Human-on-a-Chip Autoimmune Disease Model of Myasthenia Gravis for Development of Therapeutics
Source: Front Cell Dev Biol. 2021 Nov 22;9:745897. doi: 10.3389/fcell.2021.745897 (PMC8645836; doi:10.3389/fcell.2021.745897)

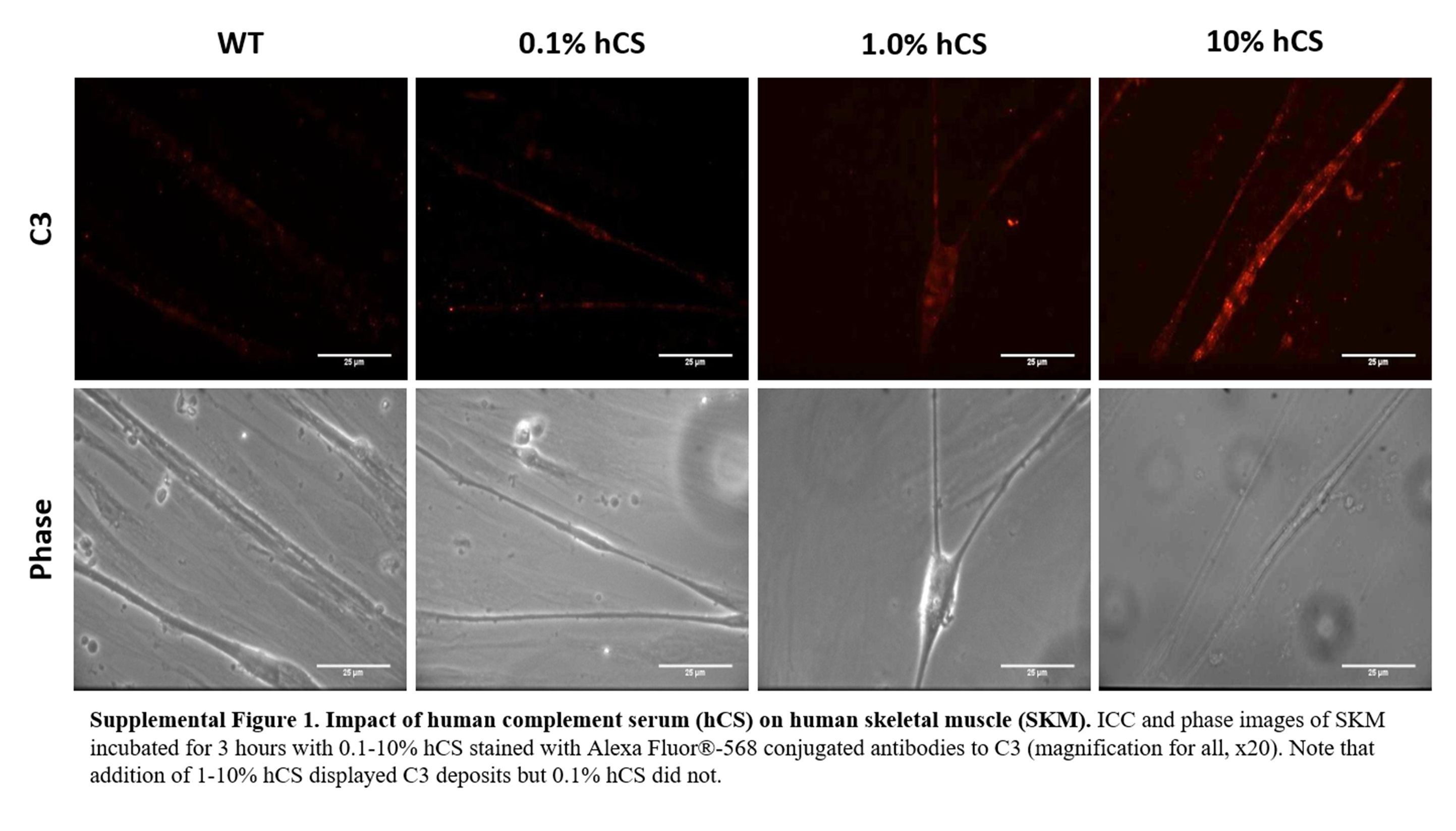

Supplement: Supplementary file 1 [file Image_1.tif]
